# Implementing electronic health records in hospitals: a systematic literature review

**DOI:** 10.1186/1472-6963-14-370

**Published:** 2014-09-04

**Authors:** Albert Boonstra, Arie Versluis, Janita F J Vos

**Affiliations:** Faculty of Economics and Business, University of Groningen, Groningen, The Netherlands; Deloitte Consulting, Amsterdam, The Netherlands

## Abstract

**Background:**

The literature on implementing Electronic Health Records (EHR) in hospitals is very diverse. The objective of this study is to create an overview of the existing literature on EHR implementation in hospitals and to identify generally applicable findings and lessons for implementers.

**Methods:**

A systematic literature review of empirical research on EHR implementation was conducted. Databases used included Web of Knowledge, EBSCO, and Cochrane Library. Relevant references in the selected articles were also analyzed. Search terms included Electronic Health Record (and synonyms), implementation, and hospital (and synonyms). Articles had to meet the following requirements: (1) written in English, (2) full text available online, (3) based on primary empirical data, (4) focused on hospital-wide EHR implementation, and (5) satisfying established quality criteria.

**Results:**

Of the 364 initially identified articles, this study analyzes the 21 articles that met the requirements. From these articles, 19 interventions were identified that are generally applicable and these were placed in a framework consisting of the following three interacting dimensions: (1) EHR context, (2) EHR content, and (3) EHR implementation process.

**Conclusions:**

Although EHR systems are anticipated as having positive effects on the performance of hospitals, their implementation is a complex undertaking. This systematic review reveals reasons for this complexity and presents a framework of 19 interventions that can help overcome typical problems in EHR implementation. This framework can function as a reference for implementers in developing effective EHR implementation strategies for hospitals.

## Background

In recent years, Electronic Health Records (EHRs) have been implemented by an ever increasing number of hospitals around the world. There have, for example, been initiatives, often driven by government regulations or financial stimulations, in the USA [1], the United Kingdom [2] and Denmark [3]. EHR implementation initiatives tend to be driven by the promise of enhanced integration and availability of patient data [4], by the need to improve efficiency and cost-effectiveness [5], by a changing doctor-patient relationship toward one where care is shared by a team of health care professionals [5], and/or by the need to deal with a more complex and rapidly changing environment [6].

EHR systems have various forms, and the term can relate to a broad range of electronic information systems used in health care. EHR systems can be used in individual organizations, as interoperating systems in affiliated health care units, on a regional level, or nationwide [1, 2]. Health care units that use EHRs include hospitals, pharmacies, general practitioner surgeries, and other health care providers [7].

The implementation of hospital-wide EHR systems is a complex matter involving a range of organizational and technical factors including human skills, organizational structure, culture, technical infrastructure, financial resources, and coordination [8, 9]. As Grimson et al. [5] argue, implementing information systems (IS) in hospitals is more challenging than elsewhere because of the complexity of medical data, data entry problems, security and confidentiality concerns, and a general lack of awareness of the benefits of Information Technology (IT). Boonstra and Govers [10] provide three reasons why hospitals differ from many other industries, and these differences might also affect EHR implementations. The first reason is that hospitals have multiple objectives, such as curing and caring for patients, and educating new physicians and nurses. Second, hospitals have complicated and highly varied structures and processes. Third, hospitals have a varied workforce including medical professionals who possess high levels of expertise, power, and autonomy. These distinct characteristics justify a study that focuses on the identification and analysis of the findings of previous studies on EHR implementation in hospitals.

## Study aim, theoretical framework, and terminology

In dealing with the complexity of EHR implementation in hospitals, it is helpful to know which factors are seen as important in the literature and to capture the existing knowledge on EHR implementation in hospitals. As such, the objective of this research is to identify, categorize, and analyze the existing findings in the literature on EHR implementation processes in hospitals. This could contribute to greater insight into the underlying patterns and complex relationships involved in EHR implementation and could identify ways to tackle EHR implementation problems. In other words, this study focusses on the identification of factors that determine the progress of EHR implementation in hospitals. The motives behind implementing EHRs in hospitals and the effects on performance of implemented EHR systems are beyond the scope of this paper.

To our knowledge, there have been no systematic reviews of the literature concerning EHR implementation in hospitals and this article therefore fills that gap. Two interesting related review studies on EHR implementation are Keshavjee et al. [11] and McGinn et al. [12]. The study of Keshavjee et al. [11] develops a literature based integrative framework for EHR implementation. McGinn et al. [12] adopt an exclusive user perspective on EHR and their study is limited to Canada and countries with comparable socio-economic levels. Both studies are not explicitly focused on hospitals and include other contexts such as small clinics and national or regional EHR initiatives.

This systematic review is explicitly focused on hospital-wide, single hospital EHR implementations and identifies empirical studies (that include collected primary data) that reflect this situation. The categorization of the findings from the selected articles draws on Pettigrew’s framework for understanding strategic change [13]. This model has been widely applied in case study research into organizational contexts [14], as well as in studies on the implementation of health care innovations [15]. It generates insights by analyzing three interactive dimensions – *context*, *content*, and *process* – that together shape organizational change. Pettigrew’s framework [13] is seen as applicable because implementing an EHR artefact is an organization-wide effort. This framework was specifically selected for its focus on organizational change, its ease of understanding, and its relatively general dimensions allowing a broad range of findings to be included. The framework structures and focusses the analysis of the findings from the selected articles.

**Figure 1:**
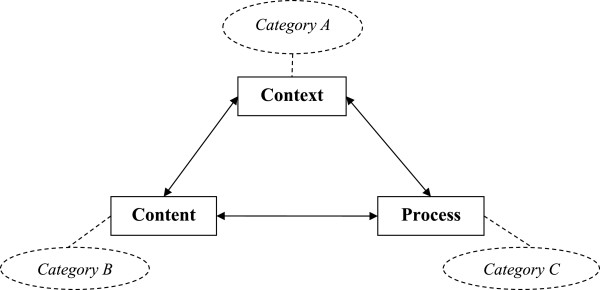
**Pettigrew**’**s framework** [13]**]** **and the corresponding categories.**

An organization’s context can be divided into internal and external components. External context refers to the social, economic, political, and competitive environments in which an organization operates. The internal context refers to the structure, culture, resources, capabilities, and politics of an organization. The content covers the specific areas of the transformation under examination. In an EHR implementation, these are the EHR system itself (both hardware and software), the work processes, and everything related to these (e.g. social conditions). The process dimension concerns the processes of change, made up of the plans, actions, reactions, and interactions of the stakeholders, rather than work processes in general. It is important to note that Pettigrew [13] does not see strategic change as a rational analytical process but rather as an iterative, continuous, multilevel process. This highlights that the outcome of an organizational change will be determined by the context, content, and process of that change. The framework with its three categories, shown in Figure 1, illustrates the conceptual model used to categorize the findings of this systematic literature review.

In the literature, several terms are used to refer to electronic medical information systems. In this article, the term Electronic Health Record (EHR) is used throughout. Commonly used terms identified by ISO (the International Organization for Standardization) [16] plus another not identified by ISO are outlined below and used in our search. ISO considers Electronic Health Record (EHR) to be an overall term for “*a repository of information regarding the health status of a subject of care*, *in computer processable form*” [16], p. 13. ISO uses different terms to describe various types of EHRs. These include Electronic Medical Record (EMR), which is similar to an EHR but restricted to the medical domain. The terms Electronic Patient Record (EPR) and Computerized Patient Record (CPR) are also identified. Häyrinen et al. [17] view both terms as having the same meaning and referring to a system that contains clinical information from a particular hospital. Another term seen is Electronic Healthcare Record (EHCR) which refers to a system that contains all the available health information on a patient [17] and can thus be seen as synonymous with EHR [16]. A term often found in the literature is Computerized Physician Order Entry (CPOE). Although this term is not mentioned by ISO [16] or by Häyrinen et al. [17], we included CPOE for three reasons. First, it is considered by many to be a key hospital-wide function of an EHR system e.g. [8, 18]. Second, from a preliminary analysis of our initial results, we found that, from the perspective of the implementation process, comparable issues and factors emerged from both CPOEs and EHRs. Third, the implementation of a comprehensive electronic medical record requires physicians to make direct order entries [19]. Kaushal et al. define a CPOE as “*a variety of computer*-*based systems that share the common features of automating the medication ordering process and that ensure standardized*, *legible*, *and complete orders*” [18], p. 1410. Other terms found in the literature were not included in this review as they were considered either irrelevant or too broadly defined. Examples of such terms are Electronic Client Record (ECR), Personal Health Record (PHR), Digital Medical Record (DMR), Health Information Technology (HIT), and Clinical Information System (CIS).

## Methods

### Search strategies

In order for a systematic literature review to be comprehensive, it is essential that all terms relevant to the aim of the research are covered in the search. Further, we need to include relevant synonyms and related terms, both for electronic medical information systems and for hospitals. By adding an * to the end of a term, the search engines pick out other forms, and by adding “ “ around words one ensures that only the complete term is searched for. Further, by including a ? as a wildcard character, every possible combination is included in the search.

The search used three categories of keywords. The first category included the following terms as approximate synonyms for hospital: “hospital*”, “healthcare”, and “clinic*”. The second category concerned implementation and included the term “implement*”. For the third category, electronic medical information systems, the following search terms were used: “Electronic Health Record*”, “Electronic Patient Record*”, “Electronic Medical Record*”, “Computeri?ed Patient Record*”, “Electronic Healthcare Record*”, “Computeri?ed Physician Order Entry”.

This relatively large set of keywords was necessary to ensure that articles were not missed in the search, and required a large number of search strategies to cover all those keywords. As we were seeking papers about the *implementation* of *electronic medical information systems* in *hospitals*, the search strategies included the terms shown in Table 1.

**Table 1:** **Overview of the search strategies**

Search strategy	Terms used**
[1]	“Electronic Health Record*” + implement* + hospital*
[2]	“Electronic Health Record*” + implement* + “health care”
[3]	“Electronic Health Record*” + implement* + clinic*
[4]	“Electronic Patient Record*” + implement* + hospital*
[5]	“Electronic Patient Record*” + implement* + “health care”
[6]	“Electronic Patient Record*” + implement* + clinic*
[7]	“Electronic Medical Record*” + implement* + hospital*
[8]	“Electronic Medical Record*” + implement* + “health care”
[9]	“Electronic Medical Record*” + implement* + clinic*
[10]	“Computeri?ed Patient Record*” + implement* + hospital*
[11]	“Computeri?ed Patient Record*” + implement* + “health care”
[12]	“Computeri?ed Patient Record*” + implement* + clinic*
[13]	“Electronic Health Care Record*” + implement* + hospital*
[14]	“Electronic Health Care Record*” + implement* + “health care”
[15]	“Electronic Health Care Record*” + implement* + clinic*
[16]	“Computeri?ed Physician Order Entry” + implement* + hospital*
[17]	“Computeri?ed Physician Order Entry” + implement* + “health care”
[18]	“Computeri?ed Physician Order Entry” + implement* + clinic*

**As suggested by the referees of this paper, *we also used the terms* “introduc*” (*instead of* “implement*”) *and* “*provider*” (*instead of physician*, *as part of CPOE*). *Each of these two searches yielded one additional article*.

The following three search engines were chosen based on their relevance to the field and their accessibility by the researcher: Web of knowledge, EBSCO, and The Cochrane Library. Most search engines use several databases but not all of them were relevant for this research as they serve a wide range of fields. Appendix A provides an overview of the databases used. The reference lists included in articles that met the selection criteria were checked for other possibly relevant studies that had not been identified in the database search.

The articles identified from the various search strategies had to be academic peer-reviewed articles if they were to be included in our review. Further, they were assessed and had to satisfy the following criteria to be included: (1) written in English, (2) full text available online, (3) based on primary empirical data, (4) focused on hospital-wide EHR implementation, and (5) meeting established quality criteria. A long list of abstracts was generated, and all of them were independently reviewed by two of the authors. They independently reviewed the abstracts, eliminated duplicates and shortlisted abstracts for detailed review. When opinions differed, a final decision over inclusion was made following a discussion between the researchers.

### Data analysis

The quality of the articles that survived this filtering was assessed by the first two authors using the Standard Quality Assessment Criteria for Evaluating Primary Research Papers [18]. In other words, the quality of the articles was jointly assessed by evaluating whether specific criteria had been addressed, resulting in a rating of 2 (fully addressed), 1 (partly addressed), or 0 (not addressed) for each criteria. Different questions are posed for qualitative and quantitative research and, in the event of a mixed-method study, both questionnaires were used. Papers were included if they received at least half of the total possible points, admittedly a relatively liberal cut-off point given comments in the Standard Quality Assessment Criteria for Evaluating Primary Research Papers [20].

The next step was to extract the findings of the reviewed articles and to analyze these with the aim of reaching general findings on the implementation of EHR systems in hospitals. Categorizing these general findings can increase clarity. The earlier introduced conceptual model, based on Pettigrew’s framework for understanding strategic change, includes three categories: context (A), content (B), and process (C). As our review is specifically aimed at identifying findings related to the implementation process, possible motives for introducing such a system, as well as its effects and outcomes, are outside its scope. The authors held frequent discussions between themselves to discuss the meaning and the categorization of the general findings.

## Results

### Paper selection

Applying the 18 search strategies listed in Table 1 with the various search engines resulted in 364 articles being identified. The searches were carried out on 12 March 2013 for search strategies 1–15 and on 18 April 2013 for search strategies 16–18. The latter three strategies were added following a preliminary analysis of the first set of results which highlighted several other terms and descriptions for information technology in health care. Not surprisingly, many duplicates were included in the 364 articles, both within and between search engines. Using the Refworks functions for identifying exact and close duplicates, 160 duplicates were found. However, this procedure did not identify all the duplicates present and the second author carried out a manual check that identified an additional 23 duplicates. When removing duplicates, we retained the link to the first search engine that identified the article and, as the Web of Knowledge was the first search engine used, most articles appear to have stemmed from this search engine. This left 181 different articles which were screened on title and abstract to check whether they met the selection criteria. When this was uncertain, the contents of the paper were further investigated. This screening resulted in just 13 articles that met all the selection criteria. We then performed two additional checks for completeness. First, checking the references of these articles identified another nine articles. Second, as suggested by the referees of this paper, we also used the term “introduc*” instead of “implement*”, together with the other two original categories of terms, and the term “provider” instead of “physician”, as part of CPOE. Each of these two searches identified one additional article (see Table 1). Of these resulting 24 articles, two proved to be almost identical so one was excluded, resulting in 23 articles for a final quality assessment.

**Figure 2:**
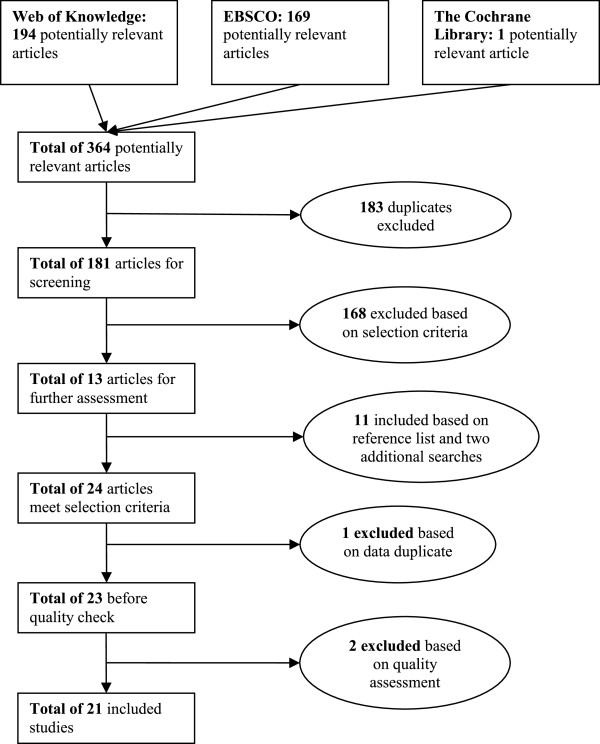
**Selection procedure.**

The results of the quality assessment can be found in Appendix B. The results show that two articles failed to meet the quality threshold and so 21 articles remained for in-depth analysis. Figure 2 displays the steps taken in this selection procedure.

To provide greater insight into the context and nature of the 21 remaining articles, an overview is provided in Table 2. All the studies except one were published after 2000. This reflects the recent increase in effort to implement organization-wide information systems, such as EHR systems, and also increasing incentives from governments to make use of EHR systems in hospitals. Of the 21 studies, 14 can be classified as qualitative, 6 as quantitative, and 1 as a mixed-method study. Most studies were conducted in the USA, with eight in various European countries. Teaching and non-teaching hospitals are almost equally the subject of inquiry, and some researchers have focused on specific types of hospitals such as rural, critical access, or psychiatric hospitals. Ten of the articles were in journals with a five-year impact factor in the Journal Citation Reports 2011 database. There is a huge difference in the number of citations but one should never forget that newer studies have had fewer opportunities to be cited.

**Table 2:** **Overview of studies included in the systematic literature review**

Author	Country/region	Main objective of study	Type of research	Data collection	Participants (sample size, response rate)	Hospital type	Impact factor*	Citations**
Aarts et al. [21]	The Netherlands	To examine the three theoretical aspects (social process, emergent change, socially negotiated judgments) to understand the implementation process.	Qualitative	Semi-structured interviews, observations, document analysis	10 members of the project team from different disciplines	Teaching hospital	4.329	194
Aarts & Berg [22]	The Netherlands	To understand the outcomes of CPOE implementation using a heuristic model and to identify factors that determine successful implementation.	Qualitative	Open interviews, observations, document analysis	25 interviews with project team members, physicians, nurses, technical and clerical personnel	Teaching hospital & regional hospital	1.090	47
Ash et al. [23]	USA/Virginia, Washington, California	To find out how some hospitals had successfully implemented POE.	Quantitative and Qualitative	Survey, semi-structured interviews, focus groups, observations	Quantitative: 1000 hospitals (37% response rate) Qualitative: 32 interviews with physicians, nurses, pharmacists, IT-staff, administrators	quantitative : 1000 hospitals qualitative: 2 teaching hospitals, 2 community hospitals	-	37
Ash et al. [24]	USA/Virginia, Washington, California	To describe perceptions of POE held by diverse professionals at both teaching and nonteaching sites where POE has been successfully implemented.	Qualitative	Semi-structured interviews, focus groups, observations	Physicians, administrators, and information technology personnel	2 teaching hospitals, 2 community hospitals	4.329	160
Boyer et al. [25]	France	To examine health care professionals’ opinions on the critical events (opportunities and barriers) surrounding EMR implementation	Qualitative	Semi-structured interviews	115 psychiatrists, nurses, psychologists and social assistants, secretaries and administrative professionals	Psychiatric teaching hospital	0.420	0
Cresswell et al. [26]	United Kingdom	To explore how EHR has shaped professional practice and what consequences these changes had for organizational functioning, record keeping and patient care.	Qualitative	Semi-structured interviews, observations, documents	66 users and other hospital staff,	3 hospitals, 1 acute setting, 1 community and mental health.	-	13
Ford et al. [27]	USA	To assess complete versus incomplete HIT implementation levels among U.S. hospitals in light of the various technology adoption strategies employed and to discuss the implications with respect to meaningful use for hospitals that have adopted the different HIT strategies.	Quantitative	Survey	1,814 hospitals	All kinds of hospitals	-	13
Gastaldi et al. [28]	Italy	To examine how hospital performance can be improved by enhancing and balancing knowledge exploration and exploitation capabilities through the development of an EMR.	Qualitative	Interviews, archival data	27 interviews in three hospitals	3 hospitals, 2 teaching and 1 non-teaching	-	2
Houser & Johnson [29]	USA/Alabama	1. To determine the status of implementation of EHRs in hospitals in the state of Alabama; 2. To assess the factors that are driving the decision making for implementation of EHRs; and 3. To assess the perceptions of HIM professionals of the benefits, barriers, and risks that are associated with implementation of EHRs.	Quantitative	Survey	131 directors in health information management, 69% response rate	Members of the Alabama Hospital Association	-	19
Jaana et al. [30]	USA/Iowa	To present an overview of clinical information systems (IS) in hospitals and to analyze the level of electronic medical records (EMR) implementation in relation to clinical IS capabilities and organizational characteristics.	Quantitative	Survey	116 CEOs or CIOs, 84% response rate	Nonfederal hospitals	-	3
Katsma et al. [31]	The Netherlands	To contribute to the developments in method engineering, which promises a better participation of the user.	Qualitative	Interviews	12 people, being supported sponsor, process owner or key-user	4 hospitals	-	4
Ovretveit et al. [32]	Sweden	To describe and assess an implementation in one hospital and analyze this in relation to factors suggested by previous research to be important for successful implementation as well as in relation to a published USA case study, which used similar methods.	Qualitative	Interviews	30 persons, project leaders, supervisors, heads of division and clinics, instructor, nurses, physicians, and doctor secretary	Teaching hospital	2.480	86
Poon et al. [33]	USA	To provide more insight into the challenges to CPOE implementation.	Qualitative	Interviews	52 CIOs/CFOs/CMOs and senior managers from 26 hospitals (46 hospitals were contacted: 57% response rate	Both teaching and non-teaching hospitals	3.748	269
Rivard et al. [34]	Canada	To propose a substantive theory – a theory developed for a particular area of inquiry (Gregor, 2006) – to provide an organizational culture-based explanation of the level of difficulty of a CIS implementation and of the implementation practices that can help reduce the level of difficulty of this process.	Qualitative	Interviews	43 people, physicians, nurses, and administrators	3 hospitals, 2 teaching and 1 community hospital	2.654	9
Scott et al. [35]	USA/Hawaii	To examine users’ attitudes to implementation of an electronic medical record system in Kaiser Permanente Hawaii.	Qualitative	Interviews	26 senior physicians, managers and project team members	One hospital, 4 clinics	13.511	174
Simon et al. [36]	USA/Massachusetts	To identify attitudes, behaviors and experiences that would constitute useful lessons for other hospitals embarking on CPOE implementation	Qualitative	Interviews, observations	24 physicians, nurses and pharmacists	5 community hospitals	-	2
Takian et al. [37]	England	To report on a case study of the implementation of an EHR (RiO) into a mental health setting delivered though the NPfIT and analyzed using our adapted ‘socio technical changing framework’.	Qualitative	Interviews, observations, document analysis	48 interviews with senior managers, implementation team members, healthcare practitioners	Mental health hospital	2.254	0
Ward et al. [38]	USA	To examine the impact of clinical information system implementation on nurses’ perceptions of workflow and patient care throughout the implementation process.	Quantitative	Survey	705 nurses	Rural hospital	-	3
Ward et al. [39]	USA	To examine staff perceptions of patient care quality and the processes before and after implementation of a comprehensive clinical information system (CIS) in critical access hospitals (CAHs).	Quantitative	Survey	840 nurses, providers, and other clinical staff	Critical access hospitals	2.540	0
Weir et al. [19]	USA/Utah	To identify factors that discriminate successful from non-successful implementation of OE/RR 2.5 in order to prepare for the next version.	Quantitative	Survey	52 medical administration staff, administrators, support staff, users (ward clerks, physicians, and nurses), and physician opinion leaders (92 received survey, thus 57% response rate)	6 hospitals	-	29
Yoon-Flannery et al. [40]	USA/New York	To determine pre-implementation perspectives of institutional, practice and vendor leadership regarding best practice for implementation of two ambulatory electronic health records (EHRs) at an academic institution.	Qualitative	Interviews	31 interviews with institutional leaders, practice leaders and vendor leaders.	Teaching hospital	-	25

*The 5-*year impact factor based on the Journal Citation Reports 2011 is used in this table*.

**The number of citations is identified using scholar.google.nl.

### Theoretical perspectives of reviewed articles

In research, it is common to use theoretical frameworks when designing an academic study [41]. Theoretical frameworks provide a way of thinking about and looking at the subject matter and describe the underlying assumptions about the nature of the subject matter [42]. By building on existing theories, research becomes focused in aiming to enrich and extend the existing knowledge in that particular field [42]. To provide a more thorough understanding of the selected articles, their theoretical frameworks, if present, are outlined in Table 3.

**Table 3:** **Overview of the theoretical frameworks used in the included studies**

Author	Theoretical framework
Aarts et al. [21]	Three theoretical aspects: 1) sociotechnical approach, 2) emergent change with an unpredictable outcome, and 3) “success” and “failure” are socially negotiated judgments and is determined by the fit between work processes and information technology.
Aarts & Berg [22]	A model on success or failure of information systems with four variables: (1) information system, (2) support base, (3) medical work practices, and (4) hospital organization. Successful implementation of an information system (1) is defined as the capability to create a support base (2) for the change of (medical) work practices (3) induced by the system (4).
Ash et al. [23]	None
Ash et al. [24]	None
Boyer et al. [25]	None
Cress-well et al. [26]	Study draws on Actor-Network Theory, which helps to investigate how the centrally procured EHR has plays an active role in shaping social relationships.
Ford et al. [27]	HIT adoption strategies: (1) Single-vendor strategy, (2) Best of Breed strategy, and (3) Best of Suite strategy.
Gastaldi et al. [28]	The notion that the capability of any organization to create sustainable organizational value not only resides in the ownership of knowledge assets guaranteeing the present competitive advantage (knowledge exploitation), but also in the ability to understand and govern the continuous development of knowledge assets necessary to renew its organizational capabilities (knowledge exploitation).
Houser & Johnson [29]	None
Jaana et al. [30]	None
Katsma et al. [31]	IT implementation success is determined by quality (relevance) times acceptation (participation). Relevance is defined as the degree to which the user expects that the IT system will solve his problems or help to realize his actually relevant goals. Participation of employees is perceived to increase their acceptation of the IT system. Effectiveness of participation is moderated by organizational receptiveness, individual ego development, and knowledge availability.
Ovretveit et al. [32]	None
Poon et al. [33]	None
Rivard et al. [34]	A culture-based explanation of the level of difficulty of a CIS implementation, using an integration perspective (basic assumptions are shared among the members of the collective), a differentiation perspective (subgroups within a collective have inconsistent interpretations), and a fragmentation perspective (members within a collective sometimes manifest multiple interpretations, irrespective their subgroup).
Scott et al. [35]	None
Simon et al. [36]	None
Takian et al. [37]	A sociotechnical framework as identified by Aarts et al. (2004), underscoring the emerging nature of change.
Ward et al. [38]	None
Ward et al. [39]	None
Weir et al. [19]	None
Yoon-Flannery et al. [40]	None

It is striking that no specific theoretical frameworks have been used in the research leading to 13 of the 21 selected articles. Most articles simply state their objective as gaining insight into certain aspects of EHR implementation (as shown in Table 1) and do not use a particular theoretical approach to identify and categorize findings. As such, these articles add knowledge to the field of EHR implementation but do not attempt to extend existing theories.

Aarts et al. [21] introduce the notion of the sociotechnical approach: emphasizing the importance of focusing both on the social aspects of an EHR implementation and on the technical aspects of the system. Using the concept of emergent change, they argue that an implementation process is far from linear and predictable due to the contingencies and the organizational complexity that influences the process. A sociotechnical approach and the concept of emergent change are also included in the theoretical framework of Takian et al. [37]. Aarts et al. [21] elaborate on the sociotechnical approach when stating that the fit between work processes and the information technology determines the success of the implementation. Aarts and Berg [22] introduce a model of success or failure in information system implementation. They see creating synergy among the medical work practices, the information system, and the hospital organization as necessary for implementation, and argue that this will only happen if sufficient people accept a change in work practices. Cresswell et al.’s study [26] is also influenced by sociotechnical principles and draws on Actor-Network Theory. Gastaldi et al. [28] perceive Electronic Health Records as knowledge management systems and question how such systems can be used to develop knowledge assets. Katsma et al. [31] focus on implementation success and elaborate on the notion that implementation success is determined by system quality and acceptance through participation. As such, they adopt more of a social view on implementation success rather than a sociotechnical approach. Rivard et al. [34] examine the difficulties in EHR implementation from a cultural perspective. They not only view culture as a set of assumptions shared by an entire collective (an integration perspective) but also expect subcultures to exist (a differentiation perspective), as well as individual assumptions not shared by a specific (sub-) group (fragmentation perspective). Ford et al. [27] focus on an entirely different topic and investigate the IT adoption strategies of hospitals using a framework that identifies three strategies. These are the single-vendor strategy (in which all IT is purchased from a single vendor), the best-of-breed strategy (integrating IT from multiple vendors), and the best-of-suit strategy (a hybrid approach using a focal system from one vendor as the basis plus other applications from other vendors).

To summarize, the articles by Aarts et al. [21], Aarts and Berg [22], Cresswell et al. [26], and Takian et al. [37] apply a sociotechnical framework to focus their research. Gastaldi et al. [28] see EHRs as a means to renew organizational capabilities. Katsma et al. [31] use a social framework by focusing on the relevance of an IT system as perceived by the user and the participation of users in the implementation process. Rivard et al. [34] analyze how organizational cultures can be receptive to EHR implementation. Ford et al. [27] look at adoption strategies, leading them to focus on the selection procedure for Electronic Health Records. The 13 other studies did not use an explicit theoretical lens in their research.

### Implementation-related findings

The process of categorization started by assessing whether a specific finding from a study should be placed in Category A, B, or C. Thirty findings were placed in Category A (context), 31 in Category B (content), and 66 in Category C (process). Comparing and combining the specific findings resulted in several general findings within each category. The general findings are each given a code (category character plus number) and the related code is indicated alongside each specific finding in Appendix C. Findings that were only seen in one article, and thus were lacking support, were discarded.

### Category A - context

The context category of an EHR implementation process includes both internal variables (such as resources, capabilities, culture, and politics) and external variables (such as economic, political, and social variables). Six general findings were identified, all but one related to internal variables. An overview of the findings and corresponding articles can be found in Table 4. The lack of general findings related to external variables reflects our decision to exclude the underlying reasons (e.g. political or social pressures) for implementing an EHR system from this review. Similarly, internal findings related to aspects such as perceived financial benefits or improved quality of care, are outside our scope.

**Table 4:** **Category A** - **Context findings**

General finding	Finding code	Article numbers
Large (or system-affiliated), urban, not-for-profit, and teaching hospitals are more likely to have implemented an EHR system due to having greater financial capabilities, a greater change readiness, and less focus on profit.	A1	27/29/30/32
EHR implementation requires the selection of a mature vendor who is committed to providing a system that fits the hospital’s specific needs.	A2	28/32/33
The presence of hospital staff with previous experience of Health Information Technology increases the likelihood of EHR implementation as less uncertainty is experienced by the end-users.	A3	19/29/32/37/38
An organizational culture that supports collaboration and teamwork fosters EHR implementation success because trust between employees is higher.	A4	23/24/25/35
EHR implementation is most likely in an organization with little bureaucracy and considerable flexibility as changes can be rapidly made.	A5	19/25
EHR system implementation is difficult because cure and care activities must be ensured at all times.	A6	28/34/39

#### A1: Large (or system-affiliated), urban, not-for-profit, and teaching hospitals are more likely to have implemented an EHR system due to having greater financial capabilities, a greater change readiness, and less focus on profit

The research reviewed shows that larger or system-affiliated hospitals are more likely to have implemented an EHR system, and that this can be explained by their easier access to the large financial resources required. Larger hospitals have more financial resources than smaller hospitals [30] and system-affiliated hospitals can share costs [27]. Hospitals situated in urban areas more often have an EHR system than rural hospitals, which is attributed to less knowledge of EHR systems and less support from medical staff in rural hospitals [29]. The fact that not-for-profit hospitals more often have an EHR system fully implemented and teaching hospitals slightly more often than private hospitals is attributed to the latter’s more wait-and-see approach and the more progressive change-ready nature of public and teaching hospitals [27, 32].

#### A2: EHR implementation requires the selection of a mature vendor who is committed to providing a system that fits the hospital’s specific needs

Although this finding is not a great surprise, it is relevant to discuss it further. A hospital selecting its own vendor can ensure that the system will match the specific needs of that hospital [32]. Further, it is important to deal with a vendor that has proven itself on the EHR market with mature and successful products. The vendor must also be able to identify hospital workflows and adapt its product accordingly, and be committed to a long-term trusting relationship with the hospital [33]. With this in mind, the initial price of the system should not be the overriding consideration: the organization should be willing to avoid purely cost-oriented vendors [28], as costs soon mount if problems arise.

#### A3: The presence of hospital staff with previous experience of health information technology increases the likelihood of EHR implementation as less uncertainty is experienced by the end-users

In order to be able to work with an EHR system, users must be capable of using information technology such as computers and have adequate typing skills [19, 32]. Knowledge of, and previous experience with, EHR systems or other medical information systems reduces uncertainty and disturbance for users, and this results in a more positive attitude towards the system [29, 32, 37, 38].

#### A4: An organizational culture that supports collaboration and teamwork fosters EHR implementation success because trust between employees is higher

The influence of organizational culture on the success of organizational change is addressed in almost all the popular approaches to change management, as well as in several of the articles in this literature review. Ash et al. [23, 24] and Scott et al. [35] highlight that a strong culture with a history of collaboration, teamwork, and trust between different stakeholder groups minimizes resistance to change. Boyer et al. [25] suggest creating a favorable culture that is more adaptive to EHR implementation. However, creating a favorable culture is not necessarily easy: a comprehensive approach including incentives, resource allocation, and a responsible team was used in the example of Boyer et al. [25].

#### A5: EHR implementation is most likely in an organization with little bureaucracy and considerable flexibility as changes can be rapidly made

A highly bureaucratic organizational structure hampers change: it slows the process and often leads to inter-departmental conflict [19]. Specifically, appointing a multidisciplinary team to deal with EHR-related issues can prevent conflict and stimulate collaboration [25].

#### A6: EHR system implementation is difficult because cure and care activities must be ensured at all times

During the process of implementing an EHR system, it is of the utmost importance that all relevant information is always available [28, 34, 39]. Ensuring the continuity of quality care while implementing an EHR system is difficult and is an important distinction from many other IT implementations.

### Category B - content

The content of the EHR implementation process consists of the EHR system and the corresponding objectives, assumptions, and complementary services. Table 5 lists the five extracted general findings. These focus on both the hardware and software of the EHR system, and its relation to work practices and privacy.

**Table 5:** **Category B** – **Content findings**

General finding	Finding code	Article numbers
Creating a fit by adapting both the technology and work practices is a key factor in the implementation of EHR.	B1	19/21/26/28/31/37
Hardware availability and system reliability in terms of speed, availability, safety, and a lack of failures, are necessary to ensure EHR use.	B2	19/24/25/29/30/35/37/40
To ensure EHR implementation, the software needs to be user-friendly with regard to ease of use, efficiency in use, and functionality.	B3	19/24/32
An EHR implementation should contain adequate safeguards for patient privacy and confidentiality.	B4	25/29/37/40
EHR implementations require a vendor who is willing to adapt its product to hospital work processes.	B5	32/33

#### B1: Creating a fit by adapting both the technology and work practices is a key factor in the implementation of EHR

This finding elaborates on the sociotechnical approach identified in the earlier section on the theories adopted in the articles. Several authors [21, 26, 31, 37] make clear that creating a fit between the EHR system and the existing work practices requires an initial acknowledgement that an EHR implementation is not just a technical project and that existing work practices will change due to the new system. By customizing and adapting the system to meet specific needs, users will become more open to using it [19, 26, 28].

#### B2: Hardware availability and system reliability, in terms of speed, availability, and a lack of failures, are necessary to ensure EHR use

In several articles, authors highlight the importance of having sufficient hardware. A system can only be used if it is available to the users, and a system will only be used if it works without problems. Ash et al. [24], Scott et al. [35], and Weir et al. [19] refer to the speed of the system as well as to the availability of a sufficient number of adequate terminals see also [40] in various locations. Systems must be logically structured [29], reliable [32], and provide safe information access [37]. Boyer et al. [25] also mention the importance of technical aspects but add that these are not sufficient for EHR implementation.

#### B3: To ensure EHR implementation, the software needs to be user-friendly with regard to ease of use, efficiency in use, and functionality

Some authors distinguish between technical availability and reliability, and the user-friendliness of the software [19, 24, 32]. They argue that it is not sufficient for a system to be available and reliable, it should also be easy and efficient in use, and provide the functionality required for medical staff to give good care. If a system fails to do this, staff will not use the system and will stick to their old ways of working.

#### B4: An EHR implementation should contain adequate safeguards for patient privacy and confidentiality

Concerns over privacy and confidentiality are recognized by Boyer et al. [25] and Houser and Johnson [29] and are considered as a barrier to EHR implementation. Yoon-Flannery et al. [40] and Takian et al. [37] also recognize the importance of patient privacy and the need to address this issue by providing training and creating adequate safeguards.

#### B5: EHR implementation requires a vendor who is willing to adapt its product to hospital work processes

A vendor must be responsive and enable the hospital to develop its product to ensure a good and usable EHR system [32, 33]. By so doing, dependence on the vendor decreases and concerns that arise within the hospital can be addressed [32]. This finding is related to A2 in the sense that an experienced, cooperative, and flexible vendor is needed to deal with the range of interest groups found in hospitals.

### Category C - process

This category refers to the actual process of implementing the EHR system. Variables considered are time, change approach, and change management. In our review, this category produced the largest number of general findings (see Table 6), as might be expected given our focus on the implementation process. EHR implementation often leads to anxiety, uncertainty, and concerns about a possible negative impact of the EHR on work processes and quality. The process findings, including leadership, resource availability, communication and participation are explicitly aimed at overcoming resistance to EHR implementation. These interventions help to create a positive atmosphere of goal directedness, co-creation and partnership.

**Table 6:** **Category C** - **Process findings**

General finding	Finding code	Article numbers
Due to their influential position, management’s active involvement and support is positively associated with EHR implementation, and also counterbalances the physicians’ medical dominance.	C1	19/24/25/32/33/34/35
Participation of clinical staff in the implementation process increases support for and acceptance of the EHR implementation.	C2	19/25/26/28/32/35/36
Training end-users and providing real-time support is important for EHR implementation success.	C3	19/29/32/36
A comprehensive implementation strategy, offering both clear guidance and room for emergent change, is needed for implementing an EHR system.	C4	19/21/25/26/28/31/37/40/36
Establishing an interdisciplinary implementation group consisting of developers, members of the IT department, and end-users fosters EHR implementation success.	C5	19/32/36
Resistance of clinical staff, in particular of physicians, is a major barrier to EHR implementation, but can be reduced by addressing their concerns.	C6	22/24/26/28/29/33/36
C7: Identifying champions among clinical staff reduces resistance.	C7	32/33/36
Assigning a sufficient number of staff and other resources to the EHR implementation process is important in adequately implementing the system.	C8	19/26/32/33/36

#### C1: Due to their influential position, management’s active involvement and support is positively associated with EHR implementation, and also counterbalances the physicians’ medical dominance

Several authors note the important role that managers play in EHR implementation. Whereas some authors refer to supportive leadership [19, 24], others emphasize that strong and active management involvement is needed [25, 32–35]. Strong leadership is relevant as it effectively counterbalances the physicians’ medical dominance. For instance, Rivard et al. [34] observe that physicians’ medical dominance and the status and autonomy of other health professionals hinder collaboration and teamwork, and that this complicates EHR implementation. Poon et al. [33] acknowledge this aspect and argue for strong leadership in order to deal with the otherwise dominant physicians. They also claim that leaders have to set an example and use the system themselves. At the same time, it is motivating that the implementation is managed by leaders who are recognized by the medical staff, for instance by head nurses and physicians or by former physicians and nurses [25, 33]. Ovretveit et al. [32] argue that it helps the implementation if senior management repeatedly declares the EHR implementation to be of the highest priority and supports this with sufficient financial and human resources. Poon et al. [33] add to this by highlighting that, especially during uncertainties and setbacks, the common vision that guides the EHR implementation has to be communicated to hospital staff. Sufficient human resources include the selection of competent and experienced project leaders who are familiar with EHR implementation. Scott et al. [35] identify leadership styles for different phases: participatory leadership is valued in selection decisions, whereas a more hierarchical leadership style is preferable in the actual implementation.

#### C2: Participation of clinical staff in the implementation process increases support for and acceptance of the EHR implementation

Participation of end-users (the clinical staff) generates commitment and enables problems to be quickly solved [25, 26, 36]. Especially because it is very unlikely that the system will be perfect for all, it is important that the clinical staff become the owner, rather than customers, of the system. Clinical staff should participate at all levels and in all steps [19, 28, 32, 36] from initial system selection onwards [35]. Ovretveit et al. [32] propose that this involvement should have an extensive timeframe, starting in the early stages of implementation, when initial vendor requirements are formulated (‘consultation before implementation’), through to the beginning of the use phase. Creating multidisciplinary work groups which determine the content of the EHR and the rules regarding the sharing of information contributes to EHR acceptance [25] and ensures realistic approaches acceptable to the clinical staff [36].

#### C3: Training end-users and providing real-time support is important for EHR implementation success

Frequently, the end-users of a new EHR system lack experience with the specific EHR system or with EHR systems in general. Although it is increasingly hard to imagine society or workplaces without IT, a large specific system, such as an EHR, still requires considerable training on how to use it properly. The importance of training is often underestimated, and inadequate training will create a barrier to EHR use [19, 29]. Consequently, adequate training, of appropriate quantity and quality, must be provided at the right times and locations [19, 32, 36]. Simon et al. [36] add to this the importance of real-time support, preferably provided by peers and super-users.

#### C4: A comprehensive implementation strategy, offering both clear guidance and room for emergent change, is needed for implementing an EHR system

Several articles highlight aspects of an EHR implementation strategy. A good strategy facilitates EHR implementation [19, 25] and consists of careful planning and preparation [36], a sustainable business plan, effective communication [28, 40] and mandatory implementation [19]. Emergent change is perceived as a key characteristic of EHR implementation in complex organizations such as hospitals [21], and this suggests an implementation approach based on a development paradigm [31], which may initially even involve parallel use of paper [26]. The notion of emergent change has been variously applied, including in the theoretical frameworks of Aarts et al. [21] and Katsma et al. [31]. These studies recognize that EHR implementation is relatively unpredictable due to unforeseen contingencies for which one cannot plan. With their emphasis on emergent change with unpredictable outcomes, Aarts et al. [21] make a case for acknowledging that unexpected and unplanned contingencies will influence the implementation process. They argue that the changes resulting from these contingencies often manifest themselves unexpectedly and must then be dealt with. Additionally, Takian et al. [37] state that it is crucial to contextualize an EHR implementation so as to be better prepared for unexpected changes.

#### C5: Establishing an interdisciplinary implementation group consisting of developers, members of the IT department, and end-users fosters EHR implementation success

In line with the arguments for management support and for the participation of clinical staff, Ovretveit et al. [32], Simon et al. [36] and Weir et al. [19] build a case for using an interdisciplinary implementation group. By having all the direct stakeholders working together, a better EHR system can be delivered faster and with fewer problems.

#### C6: Resistance of clinical staff, in particular of physicians, is a major barrier to EHR implementation, but can be reduced by addressing their concerns

Clinical staff’s attitude is a crucial factor in EHR implementation [36]. Particularly, the physicians constitute an important group in hospitals. As such, their possible resistance to EHR implementation will form a major barrier [29, 33] and may lead to workarounds [26]. Whether physicians accept or reject an EHR implementation depends on their acceptance of their work practices being transformed [22]. The likelihood of acceptance will be increased if implementers address the concerns of physicians [24, 28, 32, 33], but also of other members of clinical staff [36].

#### C7: Identifying champions among clinical staff reduces resistance

The previous finding already elaborated on clinical staff resistance and suggested reducing this by addressing their concerns. Another way to reduce their resistance is related to the process of implementation and involves identifying physician champions, typically physicians that are well respected due to their knowledge and contacts [32, 33]. Simon et al. [36] emphasize the importance of identifying champions among each stakeholder group. These champions can provide reassurance to their peers.

#### C8: Assigning a sufficient number of staff and other resources to the EHR implementation process is important in adequately implementing the system

Implementing a large EHR system requires considerable resources, including human ones. Assigning appropriate people, such as super-users [36] and a sufficient number of them to that process will increase the likelihood of success [19, 32, 33, 36]. Further, it is important to have sufficient time and financial resources [26, 32]. This finding is also relevant in relation to finding A6 (ensuring good care during organizational change).

These 19 general findings have been identified from the individual findings within the 20 analyzed articles. These findings are all related to one of the three main and interacting dimensions of the framework: six to context, five to content, and eight to process. This identification and explanation of the general findings concludes the results section of this systematic literature review and forms the basis for the discussion below.

## Discussion

This review of the existing academic literature sheds light on the current knowledge regarding EHR implementation. The 21 selected articles all originate from North America or Europe, perhaps reflecting a greater governmental attention to EHR implementation in these regions and, of course, our inclusion of only articles written in English. Two articles were rejected for quality reasons [43, 44], see Appendix B. All but one of the selected articles have been published since 2000, reflecting the growing interest in implementing EHR systems in hospitals. Eight articles built their research on a theoretical framework, four of which use the same general lens of the sociotechnical approach [21, 22, 26, 37]. Katsma et al. [31] and Rivard et al. [34] focus more on the social and cultural aspects of EHR implementation, the former on the relevance for, and participation of, users, the latter on three different cultural perspectives. Ford et al. [27] researched adoption strategies for EHR systems and Gastaldi et al. [26] consider them as a means to renew organizational capabilities. It is notable that the other reviewed articles did not use a theoretical framework to analyze EHR implementation and made no attempt to elaborate on existing theories.

A total of 127 findings were extracted from the articles, and these findings were categorized using Pettigrew’s framework for strategic change [13] as a conceptual model including the three dimensions of context, content, and process. To ensure a tight focus, the scope of the review was explicitly limited to findings related to the EHR implementation process, thus excluding the reasons for, barriers to, and outcomes of an EHR implementation.

Some of the findings require further interpretation. Contextual finding A1 relates to the demographics of a hospital. One of the assertions is that privately owned hospitals are less likely than public hospitals to invest in an EHR. The former apparently perceive the costs of EHR implementation to outweigh the benefits. This seems remarkable given that there is a general belief that information technology increases efficiency and reduces process costs, so more than compensating for the high initial investments. It is however important to note that the literature on EHR is ambivalent when it comes to efficiency; several authors record a decrease in the efficiency of work practices [25, 33, 35, 38], whereas others mention an increase [29, 31]. Finding A2 is a reminder of the importance of carefully selecting an appropriate vendor, taking into account experience with the EHR market and the maturity of their products rather than, for example, focussing on the cost price of the system. Given the huge investment costs, the price of an EHR system tends to have a major influence on vendor selection, an aspect that is also promoted by the current European tendering regulations that oblige (semi-) public institutions, like many hospitals, to select the lowest bidder, or the bidder that is economically the most preferable [45]. The finding that EHR system implementation is difficult because good medical care needs to be ensured at all times (A6) also deserves mention. Essentially, many system implementations in hospitals are different from IT implementations in other contexts because human lives are at stake in hospitals. This not only complicates the implementation process because medical work practices have to continue, it also requires a system to be reliable from the moment it is launched.

The findings regarding the content of the EHR system (Category B) highlight the importance of a suitable software product. A well-defined selection process of the software package and its associated vendor (discussed in A2) is seen as critical (B5). Selection should be based on a careful requirements analysis and an analysis of the experience and quality of the vendor. An important requirement is a sufficient degree of flexibility to customize and adapt the software to meet the needs of users and the work practices of the hospital (finding B1). At the same time the software product should challenge the hospital to rethink and improve its processes. A crucial condition for the acceptance by the diverse user groups of hospitals is the robustness of the EHR system in terms of availability, speed, reliability and flexibility (B2). This also requires adequate hardware in terms of access to computers, and mobile equipment to enable availability at all the locations of the hospital. Perceived ease of use of the system (B4) and the protection of patients’ privacy (B4) are other content factors that can make or break EHR implementation in hospitals.

The findings on the implementation process, our Category C, highlight four aspects that are commonly mentioned in change management approaches as important success factors in organizational change. The active involvement and support of management (C1), the participation of clinical staff (C2), a comprehensive implementation strategy (C4), and using an interdisciplinary implementation group (C5) correspond with three of the ten guidelines offered by Kanter et al. [46]. These three guidelines are: (1) support a strong leader role; (2) communicate, involve people, and be honest; and (3) craft an implementation plan. As the implementation of an EHR system is an organizational change process it is no surprise that these commonalities are identified in several of the analyzed articles. Three Category C findings (C2, C6, and C7) concern dealing with clinical staff given their powerful positions and potential resistance. Physicians are the most influential medical care providers, and their resistance can delay an EHR implementation [23], lead to at least some of it being dropped [21, 22, 34], or to it not being implemented at all [33]. Thus, there is ample evidence of the crucial importance of physicians’ acceptance of an EHR for it to be implemented. This means that clinicians and other key personnel should be highly engaged and motivated to contribute to EHR. Prompt feedback on requests, and high quality support during the implementation, and an EHR that clearly supports clinical work are key issues that contribute to a motivated clinical staff.

Analyzing and comparing the findings enables us to categorize them in terms of subject matter (see Table 7). By categorizing the findings in terms of subject, and by totaling the number of articles related to the individual findings on that subject, one can deduce how much attention has been given in the literature to the different topics. This analysis highlights that the involvement of physicians in the implementation process, the quality of the system, and a comprehensive implementation strategy are considered the crucial elements in EHR implementation.

**Table 7:** **Findings sorted by subject**

Subject	Related findings	Nr. of articles
Leadership and involvement in the process	C1, C2, C5, C8	10
Vendor	A2, B5	3
Implementation strategy	C4, C5,	10
Role of clinical staff (in particular the physicians)	C6, C7,	8
Users’ skills/experience	A3, C3	6
EHR system	B2, B3	8
Patient issues	A6, B4,	7
Hospital demographics	A1	4
Organizational culture	A4	4
Organizational structure	A5	2
Fit between work processes and EHR system	B1	6

Notwithstanding the useful results, this review and analysis has some limitations. Although we carefully developed and executed the search strategy, we cannot be sure that we found all the relevant articles. Since we focused narrowly on keywords, and these had to be part of an article’s title, we could have excluded relevant articles that used different terminology in their titles. Although searching the reference lists of identified articles did result in several additional articles, some relevant articles might still have been missed. Another limitation is the exclusion of publications in languages other than English. Further, the selection and categorization of specific findings, and the subsequent extraction of general findings, is subjective and depends on the interpretations of the authors, and other researchers might have made different choices. A final limitation is inherent to literature reviews in that the authors of the studies included may have had different motives and aims, and used different methods and interpretative means, in drawing their conclusions.

## Conclusions

The existing literature fails to provide evidence of there being a comprehensive approach to implementing EHR systems in hospitals that integrates relevant aspects into an ‘EHR change approach’. The literature is diffuse, and articles seldom build on earlier ones to increase the theoretical knowledge on EHR implementation, notable exceptions being Aarts et al. [21], Aarts and Berg [22], Cresswell et al. [26], and Takian et al. [37]. The earlier discussion on the various results summarizes the existing knowledge and reveals gaps in the knowledge associated with EHR implementation. The number of EHR implementations in hospitals is growing, as well as the body of literature on this subject. This systematic review of the literature has produced 19 general findings on EHR implementation, which were each placed in one of three categories. A number of these general findings are in line with the wider literature on change management, and others relate to the specific nature of EHR implementation in hospitals.

The findings presented in this article can be viewed as an overview of important subjects that should be addressed in implementing an EHR system. It is clear that EHR systems have particular complexities and should be implemented with great care, and with attention given to context, content, and process issues and to interactions between these issues. As such, we have achieved our research goal by creating a systematic review of the literature on EHR implementation. This paper’s academic contribution is in providing an overview of the existing literature with regard to important factors in EHR implementation in hospitals. Academics interested in this specific field can now more easily access knowledge on EHR implementation in hospitals and can use this article as a starting point and build on the existing knowledge. The managerial contribution lies in the general findings that can be applied as guidelines when implementing EHR in hospitals. We have not set out to provide a single blueprint for implementing an EHR system, but rather to provide guidelines and to highlight points that deserve attention. Recognizing and addressing these aspects can increase the likelihood of getting an EHR system successfully implemented.

## Appendix

### Appendix A - List of databases

This appendix provides an overview of all databases included in the used search engines. The databases in italic were excluded for the research as these databases focus on fields not relevant for the subject of EHR implementations.

#### Web of Knowledge

- 1) Web of Science
- 2) Biological Abstracts
- 3) Inspec
- 4) MEDLINE
- 5) Journal Citation Reports

#### EBSCO

- 1) Academic Search Premier
- 2) AMED - The Allied and Complementary Medicine Database
- 3) *America*: *History & Life*
- 4) *American Bibliography of Slavic and East European Studies*
- 5) *Arctic & Antarctic Regions*
- 6) *Art Full Text* (*H.W. Wilson*)
- 7) *Art Index Retrospective* (*H.W. Wilson*)
- 8) *ATLA Religion Database with ATLASerials*
- 9) Business Source Premier
- 10) CINAHL
- 11) *Communication & Mass Media Complete*
- 12) *eBook Collection* (*EBSCOhost*)
- 13) *EconLit*
- 14) ERIC
- 15) *Funk & Wagnalls New World Encyclopedia*
- 16) *GreenFILE*
- 17) *Historical Abstracts*
- 18) *L*’*Annéephilologique*
- 19) Library, Information Science & Technology Abstracts
- 20) *MAS Ultra* - *School Edition*
- 21) MEDLINE
- 22) *Military & Government Collection*
- 23) *MLA Directory of Periodicals*
- 24) *MLA International Bibliography*
- 25) *New Testament Abstracts*
- 26) *Old Testament Abstracts*
- 27) *Philosopher*’*s Index*
- 28) *Primary Search*
- 29) PsycARTICLES
- 30) *PsycBOOKS*
- 31) *PsycCRITIQUES*
- 32) Psychology and Behavioral Sciences Collection
- 33) PsycINFO
- 34) *Regional Business News*
- 35) *Research Starters* - *Business*
- 36) *RILM Abstracts of Music Literature*
- 37) SocINDEX

#### The Cochrane Library

- 1) Cochrane Database of Systematic Reviews
- 2) Cochrane Central Register of Controlled Trials
- 3) Cochrane Methodology Register
- 4) Database of Abstracts of Reviews of Effects
- 5) Health Technology Assessment Database
- 6) NHS Economic Evaluation Database
- 7) About The Cochrane Collaboration

### Appendix B - Quality assessment

The quality of the articles was assessed with the Standard Quality Assessment Criteria for Evaluating Primary Research Papers [18]. Assessment was done by questioning whether particular criteria had been addressed, resulting in a rating of 2 (completely addressed), 1 (partly addressed), or 0 (not addressed) points. Table 8 provides the overview of the scores of the articles, (per question) for qualitative studies; Table 9 for quantitative studies; and Table 10 for mixed methods studies. Articles were included if they scored 50% or higher of the total amount of points possible. Based on this assessment, two articles were excluded from the search.

**Table 8:** **Quality assessment results of qualitative studies**

Criteria qualitative studies	[21]	[22]	[24]	[25]	[26]	[28]	[31]	[32]	[33]	[34]	[35]	[36]	[37]	[40]
Question/objective sufficiently described?	2	2	2	2	2	2	2	2	1	2	1	2	1	2
Study design evident and appropriate?	2	2	2	2	2	2	2	2	2	2	2	2	2	2
Context for the study clear?	2	2	2	2	2	2	1	2	1	2	1	2	2	1
Connection to a theoretical framework/wider body of knowledge?	2	1	0	2	1	0	2	2	0	2	1	0	2	1
Sampling strategy described, relevant and justified?	0	0	2	2	2	2	1	0	1	2	1	2	2	2
Data collection methods clearly described and systematic?	1	1	2	2	2	2	1	2	2	1	1	2	1	2
Data analysis clearly described and systematic?	0	0	1	1	1	2	0	1	1	2	1	2	1	2
Use of verification procedure (s) to establish credibility?	0	2	2	0	1	1	0	0	0	0	0	1	2	0
Conclusions supported by the results?	1	2	2	2	2	0	1	2	2	2	2	2	1	2
Reflexivity of the account?	0	0	0	0	0	0	0	0	1	0	0	2	2	2
Total score/possible maximum score	**10/20**	**12/20**	**15/20**	**15/20**	**15/20**	**13/20**	**10/20**	**13/20**	**11/20**	**15/20**	**10/20**	**17/20**	**16/20**	**16/20**

**Table 9:** **Quality assessment results of quantitative studies**

Criteria quantitative studies	[27]	[29]	[30]	[44]	[38]	[39]	[19]
Question/objective sufficiently described?	2	2	2	1	2	2	2
Study design evident and appropriate?	2	2	2	1	2	2	2
Method of subject/comparison group selection or source of information/input variables described and appropriate?	1	2	1	1	2	1	2
Subject (and comparison group, if applicable) characteristics sufficiently described?	2	2	2	1	2	2	1
If interventional and random allocation was possible, was it described?	N/A	N/A	N/A	N/A	N/A	N/A	N/A
If interventional and blinding of investigators was possible, was it reported?	N/A	N/A	N/A	N/A	N/A	N/A	N/A
If interventional and blinding of subjects was possible, was it reported?	N/A	N/A	N/A	N/A	N/A	N/A	N/A
Outcome and (if applicable) exposure measure (s) well defined and robust to measurement/misclassification bias? Means of assessment reported?	0	2	1	0	2	2	2
Sample size appropriate?	2	2	2	2	2	2	2
Analytic methods described/justified and appropriate?	1	1	2	0	0	1	2
Some estimate of variance is reported for the main results?	1	0	2	0	1	1	N/A
Controlled for confounding?	2	1	2	0	1	1	N/A
Results reported in sufficient detail?	2	2	2	1	2	2	2
Conclusions supported by the results?	1	2	2	2	2	2	2
Total score/possible maximum score	**16/22**	**19/22**	**20/22**	**9/22**	**18/22**	**18/22**	**17/18**

**Table 10:** **Quality assessment results of mixed methods studies**

Qualitative criteria mixed methods studies	[23]	[43]
Question/objective sufficiently described?	1	2
Study design evident and appropriate?	2	2
Context for the study clear?	2	1
Connection to a theoretical framework/wider body of knowledge?	0	1
Sampling strategy described, relevant and justified?	1	0
Data collection methods clearly described and systematic?	1	1
Data analysis clearly described and systematic?	1	0
Use of verification procedure (s) to establish credibility?	0	0
Conclusions supported by the results?	1	1
Reflexivity of the account?	0	0
**Quantitative criteria mixed methods studies**		
Question/objective sufficiently described?	1	2
Study design evident and appropriate?	2	2
Method of subject/comparison group selection or source of information/input variables described and appropriate?	2	1
Subject (and comparison group, if applicable) characteristics sufficiently described?	1	0
If interventional and random allocation was possible, was it described?	N/A	N/A
If interventional and blinding of investigators was possible, was it reported?	N/A	N/A
If interventional and blinding of subjects was possible, was it reported?	N/A	N/A
Outcome and (if applicable) exposure measure (s) well defined and robust to measurement/misclassification bias? Means of assessment reported?	2	0
Sample size appropriate?	2	N/A
Analytic methods described/justified and appropriate?	2	0
Some estimate of variance is reported for the main results?	1	0
Controlled for confounding?	N/A	N/A
Results reported in sufficient detail?	2	0
Conclusions supported by the results?	2	1
**Total score/possible maximum score**	**26/40**	**14/38**

### Appendix C - All findings

Table 11 displays all findings from the selected articles. The category number is related to the general finding as discussed in the Results section.

**Table 11:** **Overview of all findings**

Author	Findings	Category
Ash et al. [23]	Trust between administrators and physicians seems to be a necessary ingredient tot successful implementation.	A4
Ash et al. [24]	Organizational issue fostering implementation: a strong culture	A4
Ash et al. [24]	Organizational issue fostering implementation: a history of collaboration and teamwork	A4
Boyer et al. [25]	A favorable strategic factor is creating a favorable organizational culture.	A4
Boyer et al. [25]	The establishment of a multidisciplinary team to deal with her related issues prevents conflict and stimulates collaboration.	A5
Ford et al. [27]	For-profit hospitals are half as likely to have fully implemented an EHR as their nonprofit counterparts.	A1
Ford et al. [27]	System-affiliated hospitals were 31 percent more likely than were unaffiliated facilities to have successfully implemented an EHR.	A1
Gastaldi et al. [28]	Willingness to avoid pure cost-oriented vendors.	A2
Gastaldi et al. [28]	Diffused pressures to realize the EMR as soon as possible, because physicians’ data sharing is needed.	A6
Houser & Johnson [29]	Rural hospitals are less likely to have completed implementation of an EHR system compared to urban and suburban hospitals.	A1
Houser & Johnson [29]	Government-owned or not-for-profit hospitals more often implemented a complete EHR system compared to for-profit hospitals.	A1
Houser & Johnson [29]	A perceived barrier of implementing an EHR system is the lack of knowledge of EHR systems.	A3
Jaana et al. [30]	Critical Access Hospitals (CAH) in Iowa have significantly lower EMR levels compared to non-CAHs.	A1
Jaana et al. [30]	A higher number of staffed beds and available slack resources is positively associated with higher clinical IS scores and EMR levels.	A1
Ovretveit et al. [32]	A facilitating factor in implementing an EMR system is the local hospital control of selection of the system.	A2
Ovretveit et al. [32]	A facilitating factor in implementing an EMR system is previous computer or EMR experience.	A3
Ovretveit et al. [32]	A facilitating factor in implementing an EMR system is the academic medical centre being more change ready.	A1
Poon et al. [33]	A barrier to implementing CPOE is product and vendor immaturity.	A2
Poon et al. [33]	Product and vendor immaturity can be overcome by selecting a vendor who is committed to the CPOE market.	A2
Poon et al. [33]	Product and vendor immaturity can be overcome by ensuring a long-term trusting relationship of the vendor with the hospital.	A2
Rivard et al. [34]	The difficulty of a CIS implementation is explained by quality of care.	A6
Scott et al. [35]	The organizational culture of cooperative values minimized resistance to change early on.	A4
Takian et al. [37]	In order to successfully implement an EHR stakeholders, and their computer literacy and ability to access the technology, need to be identified prior to planning to procure and implement EHR software.	A3
Ward et al. [38]	Nurses who had previous experience with EHRs at other hospitals expressed more positive views towards an EHR.	A3
Ward et al. [38]	Nurses with more years of health care experience had less favorable perceptions towards an EHR compared to nurses with less years of experience.	A3
Ward et al. [39]	The staff perceived the EHR/CPOE implementation not to have disrupted the existing care processes.	A6
Weir et al. [19]	A barrier to successful implementation of a CPOE is an uncooperative or computer phobic attitude of physicians.	A3
Weir et al. [19]	A barrier to successful implementation of a CPOE is bureaucracy preventing change and interdepartmental conflict.	A5
Weir et al. [19]	A barrier to successful implementation of a CPOE is health care providers that don’t know how to type.	A3
Weir et al. [19]	Support staff identify the barrier bureaucracy significantly more often than physicians.	A5
Aarts et al. [21]	Implementation of a CPOE is both a social process and contains technical issues, which increases complexity.	B1
Aarts et al. [21]	Creating fit between technology and work practices is a key factor for successful implementation of information systems.	B1
Ash et al. [24]	Technical/implementation issue fostering implementation: speed of the system	B2
Ash et al. [24]	Technical/implementation issue fostering implementation: the ability to group orders into order sets	B3
Ash et al. [24]	Technical/implementation issue fostering implementation: the possibility to make clinical pathways available to health care teams,	B3
Ash et al. [24]	Technical/implementation issue fostering implementation: the possibility to enter orders from remote locations.	B2
Ash et al. [24]	Organization of information issue fostering implementation: the information must be organized in a manner designed to mimic the way in which people use the information, which is generally not in a structured, hierarchical manner.	B3
Boyer et al. [25]	The technical aspects of an EMR have an important place but do not necessarily guarantee a successful implementation of EMR.	B2
Boyer et al. [25]	A barrier in implementing an EMR is less confidentiality in information sharing between patient and professional.	B4
Cresswell et al. [26]	A barrier in implementing an EHR is limited ability to customize the software.	B1
Gastaldi et al. [28]	Being able to deal with technical problems related to the customization of the system.	B1
Houser & Johnson [29]	A perceived barrier of implementing an EHR system is the lack of structured technology.	B2
Houser & Johnson [29]	Perceived barriers of implementing an EHR system are privacy and confidentiality issues.	B4
Katsma et al. [31]	Compatibility of the EPR with working processes can also be reached by changing the work processes.	B1
Ovretveit et al. [32]	A factor in implementing an EMR system is the ease of navigation, efficiency in use and accessibility of the system.	B3
Ovretveit et al. [32]	A factor in implementing an EMR system is the absence of failures	B2
Ovretveit et al. [32]	A factor in implementing an EMR system is physicians’ acceptance and implementer’s responsiveness to concerns.	B5
Poon et al. [33]	Product and vendor immaturity can be overcome by having the vendor willing to adapt its product to hospital workflow issues.	B5
Scott et al. [35]	Software design and development problems increased local resistance.	B2
Takian et al. [37]	EHR needs to be seen as a sociotechnical entity by stakeholders, ensuring a user-centered design of EHR.	B1
Takian et al. [37]	Because of the huge cultural shift an EHR brings to heavily text-based notes, healthcare practitioners must be educated and protected with regards to transparency and observing confidentiality of patient notes.	B4
Takian et al. [37]	The safety of information access to EHR systems needs to be ensured prior to and during the implementation.	B2
Weir et al. [19]	A facilitating factor associated with implementation of a CPOE is sufficient functionality of the system.	B3
Weir et al. [19]	A facilitating factor associated with l implementation of a CPOE is the ability to customize software to meet physician needs.	B1
Weir et al. [19]	A facilitating factor associated with implementation of a CPOE is adequate hardware, terminals, etc.	B2
Weir et al. [19]	A barrier to implementation of a CPOE is insufficient functionality of the software.	B3
Weir et al. [19]	A barrier to implementation of a CPOE is having an insufficient number of terminals, a too slow system, and non-portable screens.	B2
Weir et al. [19]	A barrier to implementation of a CPOE is a user-unfriendly system.	B3
Weir et al. [19]	A barrier to implementation of a CPOE is a too labor intensive program.	B3
Yoon-Flannery et al. [40]	EHR implementation best practice contains sufficient hardware, technical equipment, support and training.	B2
Yoon-Flannery et al. [40]	EHR implementation best practice contains adequate safeguards for patient privacy.	B4
Aarts et al. [21]	Emergent change is a key characteristic of implementing information systems in complex organizations.	C4
Ash et al. [24]	Organizational issue fostering implementation: supportive leadership	C1
Boyer et al. [25]	The strategy used for EMR implementation is particularly important	C4
Boyer et al. [25]	A favorable strategic factor is active involvement of the manager.	C1
Boyer et al. [25]	A favorable strategic factor is regularly assessing the views of professionals to identify problems and develop support for corrective action.	C2
Cresswell et al. [26]	Allowing intensive user involvement in software design is favorable for embedding the system of time (particularly in smaller scale implementations).	C2
Cresswell et al. [26]	Acceptance of initially parallel use of paper during the implementation.	C4
Cresswell et al. [26]	Resistance of powerful users can lead to ‘workarounds’	C6
Cresswell et al. [26]	There is time and resources available to let the users familiarize with the system.	C8
Gastaldi et al. [28]	Engagement of the whole organization in the process is crucial (both the creation as well as the maintenance).	C2
Gastaldi et al. [28]	Management of the change is crucial, particularly its initial communication.	C4
Gastaldi et al. [28]	Initial technological resistance of the physicians is a problem.	C6
Gastaldi et al. [28]	Understanding of the physicians’ necessities is important.	C6
Houser & Johnson [29]	A perceived barrier of implementing an EHR system is the lack of employee training.	C3
Katsma et al. [31]	Development paradigm implementation approaches go hand in hand with high levels of implementation.	C4
Ovretveit et al. [32]	A helping factor in implementing an EMR system is employee involvement in many different ways.	C2
Ovretveit et al. [32]	A helping factor in implementing an EMR system is leadership and support by a competent on site information technology department.	C5
Ovretveit et al. [32]	A helping factor in implementing an EMR system is decisive and full leadership backing.	C1
Ovretveit et al. [32]	A factor in implementing an EMR system is user involvement in selection and development.	C5
Ovretveit et al. [32]	A factor in implementing an EMR system is providing education at the right times, amount and quality.	C3
Ovretveit et al. [32]	A factor in implementing an EMR system is strong management support.	C1
Simon et al. [36]	The entity that manages the implementation of CPOE needs to have representation from among the staff members (front line representation).	C2
Simon et al. [36]	Training end-users is important; providing real-time support is even more important.	C3
Simon et al. [36]	CPOE implementation requires a great deal of planning and preparation in advance.	C4
Simon et al. [36]	Multi-disciplinary representation of front line users and collaboration is important for the implementation of CPOE.	C5
Simon et al. [36]	Awareness of attitudes of anxiety and fear is important in the planning of the implementation of CPOE.	C6
Simon et al. [36]	The identification and support of a champion among each user group.	C7
Simon et al. [36]	The ample presence of live, in-person support (super-users) is helpful in facilitating the CPOE implementation.	C8
Scott et al. [35]	The initial selection of the CIS was perceived to be detached from the local environment resulting in conflicting priorities between the organization and individual physicians.	C2
Scott et al. [35]	Participatory leadership was valued for selection decisions.	C1
Scott et al. [35]	Hierarchical leadership was valued for implementation.	C1
Weir et al. [19]	A facilitating factor associated with the implementation of a CPOE is knowledgeable, cheerful support from the Information Resource Management department.	C5
Weir et al. [19]	A facilitating factor associated with the implementation of a CPOE is supportive administration and chiefs of staff.	C1
Weir et al. [19]	A facilitating factor associated with the implementation of a CPOE is direct involvement of physicians.	C2
Weir et al. [19]	A facilitating factor associated with the implementation of a CPOE is a good working relationship with developers.	C5
Weir et al. [19]	A facilitating factor associated with the implementation of a CPOE is an interdisciplinary, effective implementation group.	C5
Weir et al. [19]	A facilitating factor associated with the implementation of a CPOE is a good implementation strategy.	C4
Weir et al. [19]	A facilitating factor associated with the implementation of a CPOE is support by medical administration and other allied fields.	C2
Weir et al. [19]	A facilitating factor associated with the implementation of a CPOE is mandatory implementation.	C4
Weir et al. [19]	A facilitating factor associated with the implementation of a CPOE is good training and instruction.	C3
Weir et al. [19]	A barrier to the implementation of a CPOE is inadequate training, insufficient material, and residents rotation.	C3
Weir et al. [19]	A barrier to the implementation of a CPOE is the lack of effective, cheerful Information Resource Management support.	C5
Weir et al. [19]	A barrier to the implementation of a CPOE is non-supportive section chiefs of staff.	C1
Weir et al. [19]	Support staff identifies the facilitating factor organized, interdisciplinary implementation group significantly more often than physicians.	C5
Weir et al. [19]	Physicians identify the facilitating factor support of chiefs of staff and medical administration significantly more often than support staff.	C1
Weir et al. [19]	Physicians identify the facilitating factor mandatory implementation significantly more often than support staff.	C4
Weir et al. [19]	A facilitating factor associated with successful implementation of a CPOE is having a sufficient number of people for implementation and user training.	C8
Weir et al. [19]	A barrier to successful implementation of a CPOE is insufficient personnel to adequately implement the system and train people.	C8
Weir et al. [19]	Support staff identifies the facilitating factor sufficient personnel for implementation significantly more often than physicians.	C8
Yoon-Flannery et al. [40]	EHR implementation best practice contains effective, clear communication.	C4
Yoon-Flannery et al. [40]	EHR implementation best practice contains careful planning for system migration.	C4
Yoon-Flannery et al. [40]	EHR implementation best practice contains a sustainable business plan.	C4
Aarts & Berg [22]	Accepting or rejecting an information system will depend on whether those involved in the medical work practices will accept a transformation of these practices.	C6
Ash et al. [24]	Clinical/Professional issue fostering implementation: customization and the ability to adapt POE at the local level, creating acceptance among physicians.	C6
Houser & Johnson [29]	A perceived barrier of implementing an EHR system is the lack of support from medical staff.	C6
Ovretveit et al. [32]	A facilitating factor in implementing an EMR system is having adequate people and financial resources.	C8
Poon et al. [33]	A barrier to implementing CPOE is physician and organizational resistance.	C6
Poon et al. [33]	Physician and organizational resistance can be overcome by addressing workflow concerns.	C6
Aarts et al. [21]	The implementation process of a CPOE is highly unpredictable, influenced by contingencies that were not expected nor planned for.	C4
Ovretveit et al. [32]	A factor in implementing an EMR system is having a physician champion.	C7
Poon et al. [33]	Physician and organizational resistance can be overcome by strong leadership.	C1
Poon et al. [33]	Physician and organizational resistance can be overcome by identifying physician champions.	C7
Poon et al. [33]	Physician and organizational resistance can be overcome by leveraging house staff or hospitalists.	C8
Rivard et al. [34]	The difficulty of a CIS implementation is explained by physicians’ medical dominance.	C1
Rivard et al. [34]	The difficulty of a CIS implementation is explained by other health professionals’ professional status and autonomy.	C1
Takian et al. [37]	Contextualization and taking heterogeneity across mental health settings is crucial to implement EHR initiatives, it might help identify areas in need of additional support.	C4
